# First isolation and characterization of Getah virus from cattle in northeastern China

**DOI:** 10.1186/s12917-019-2061-z

**Published:** 2019-09-05

**Authors:** Hao Liu, Xu Zhang, Li-Xia Li, Ning Shi, Xiu-tao Sun, Quan Liu, Ning-Yi Jin, Xing-kui Si

**Affiliations:** 1 School of Life Sciences and Engineering, Foshan University, Foshan, 528000 Guangdong Province China; 2 Forestry Department of Jilin Province, Jilin Wildlife Rescue and Rehabilitation Center, Changchun, 130122 Jilin Province China; 3 Honghe Animal Disease Prevention and Control Center, Mengzi, 661000 Yunnan Province China; 4 Military Veterinary Institute, Academy of Military Medical Sciences, Changchun, 130122 Jilin Province China

**Keywords:** Getah virus, Cattle, Phylogenetic analysis, qRT-PCR, Northeastern China

## Abstract

**Background:**

Getah virus (GETV) is a neglected mosquito-borne *Alphavirus* that causes pyrexia, body rash, and leg oedema in horses and foetal death and reproductive disorders in pigs. Infected animals may play a critical role in the amplification and circulation of the virus. The present study aimed to investigate GETV infection in clinically infected cattle and vector mosquito species in northeastern China.

**Results:**

Serum samples were collected from beef cattle that presented sudden onset of fever in forest grazing areas, and metagenomic sequencing was conducted, revealing 29 contigs from ten serum samples matching the GETV genome. Quantitative RT-PCR (RT-qPCR) was performed with GETV RNA from 48 beef cattle serum samples, showing that the overall prevalence of GETV in the beef cattle samples was 6.25% (3/48). Serological investigation indicated that GETV neutralizing antibodies were detected in 83.3% (40/48, 95% CI 67–100) of samples from the study region. The GETV JL1808 strain was isolated from clinically infected cattle showing fever. Sequence comparisons showed high identity with the HuN1 strain, a highly pathogenic swine epidemic isolate obtained in Hunan province in 2017, at the nucleotide level (99.5%) and at the deduced amino acid level (99.7–99.9%). The phylogenetic analysis of JL1808 clustered in Group III, and also revealed a close genetic relationship with the HuN1 strain. Additionally, about 12,000 mosquitoes were trapped in this region. The presence of GETV infection was detected in mosquitoes, suggesting that the minimum infection rate (MIR) was 1.50‰, with MIRs of 1.67‰ in *Culex pseudovishnui*, 1.60‰ in *Culex tritaeniorhynchus*, and 1.21‰ in *Anopheles sinensis*.

**Conclusions:**

To the best of our knowledge, this is the first report of GETV infection in cattle. These results demonstrated that a highly pathogenic, mosquito-borne swine GETV can infect and circulate in cattle, implying that it is necessary to conduct surveillance of GETV infection in animals in northeastern China.

**Electronic supplementary material:**

The online version of this article (10.1186/s12917-019-2061-z) contains supplementary material, which is available to authorized users.

## Background

Getah virus (GETV) belongs to the genus *Alphavirus* (family: *Togaviridae*), whose genome includes single positive-stranded RNA. These viruses are mostly arthropod-borne and are primarily transmitted by various mosquito species, including *Culex*, *Anopheles*, *Aedes*, *Armigeres*, and *Mansonia* spp*.* [1, 2]. GETV was first isolated from a *Culex* mosquito in Malaysia in 1955, and later found in a variety of mosquito species and animals [3–7]. The GETV lifecycle is similar to that of the Japanese encephalitis virus; it is transmitted via *Culex* mosquitoes and amplified in domestic pigs [8]. It can cause death in young piglets, miscarriage in pregnant sows, and mild illness in horses [9–11].

GETV is spread over a broad geographical area from Malaysia (latitude 3°N) through mainland China to Russia (latitude 60°N) and involves a wide range of hosts [5, 12]. In China, GETV was first identified in Hainan [7]. Since then, It has been reported to be widely distributed in 12 provinces, ranging between latitudes 19°N and 40°N and longitudes 97°E and 122°E including southwestern China (Yunnan Province) and northern China (Liaoning Province), and has spread rapidly and caused outbreaks in recent years [7, 9, 12–15]. The latest GETV outbreak occurred on a swine farm in Hunan, China, in June and July 2017 [9]. Serological surveys have shown that infections have occurred in multiple vertebrate species, including pigs, cattle and poultry, in Yunnan Province [16]. There is evidence that the virus can infect humans and cause fever [7] . Phylogenetic analysis of the E2 gene of GETV strains has evolved into four distinct groups [12]. GETV strains of infect animals were most clustered in Group III in China.

Up to now, there is only serological evidence supporting the presence of antibodies against GETV in beef cattle with a positive rate of 72% (23/32) in Yunnan Province in 2015 [16]. There has been no report of the isolation of GETV from cattle in Jilin province. The present study aimed to investigate the GETV infection in cattle, the vector mosquito species and the mosquito positivity rate to identify the possible mosquito vectors of GETV in the northeast forest area of China.

## Results

### Molecular and serological assays for samples

In 2018, we have collected blood samples from 48 beef cattle in the Jiaohe forest areas. RNA extracted from 10 cattle serum samples with clinical symptoms was used for random PCR amplification, followed by library preparation and viral metagenomics analysis. Metagenomic sequencing revealed 29 contigs matching the GETV genome (online Technical Additional file 1: Table S1). Moreover, a total of 48 cattle serum samples were detected by RT-qPCR and neutralizing antibody analyses. The results showed that a total of 3 sick beef cattle were positive by RT-qPCR. The GETV infection rate in beef cattle was 6.25% (3/48). GETV neutralizing antibodies were detected in 83.3% (40/48; 95% CI 67–100) of the beef cattle serum samples. Regarding GETV neutralizing antibody titres, beef cattle serum samples with titres between 1:160 and 1:320 were classified as medium-titre samples and those with titres between 1:640 and 1:1280 were classified as high-titre samples; medium-titre samples accounted for 33.3% (16/48; 95% CI 28–38) of the positive specimens, and high-titre samples accounted for 35.4% (17/48, 95% CI 16–55) of the positive specimens (Table 1). Additionally, potential mosquito vectors were collected from the studied region and tested for GETV. The results showed a MIR of 1.50‰ in mosquitoes (Table 2). We found that *Cx. pseudovishnui* and *Cx. tritaeniorhynchus* were the main vectors, and beef cattle were likely to be the hosts enabling the proliferation of GETV.

**Table 1:** Results of SN test and RT-qPCR for GETV in serum samples of cattle from Jilin, northeastern China^a^

Group^b^	Clinical symptoms	SN test (no. of samples)	RT-qPCR
1	No symptoms	<1:5 (*n* = 5)	–
2	Fever, appetite loss, and depression	1:5 (*n* = 1)	+
	Fever, appetite loss, and depression	1:5 (*n* = 1)	+
	Fever, appetite loss, and depression	1:5 (*n* = 1)	+
	Fever, appetite loss, and depression	1:10(*n* = 1)	–
	Fever, appetite loss	1:20 (*n* = 2)	–
	Appetite loss	1:40 (*n* = 3)	–
	Appetite loss	1:80 (*n* = 1)	–
3	No symptoms	1:160–1:320 (*n* = 16)	–
4	No symptoms	1:640–1:1280 (*n* = 17)	–

^a^SN, serum neutralization; RT-qPCR, quantitative reverse transcription polymerase chain reaction; −, negative;+, positive

^b^The collected samples were divided into Group 1 (<1:5), Group 2 (< 1:80), Group 3 (1:160–1:320), and Group 4 (1:640–1:1280) according to the neutralizing antibody titer > 1:5 was positive

**Table 2:** RT-qPCR to determine Getah virus in mosquito samples in northeastern China in 2018

Species	No. of mosquitoes	No. of pools (100 mosquitoes/pool)	No. Positive pools	MIR of Mosquitoes (‰)^‡^
*Culex pseudovishnui*	1200	12	2	1.67 (2/1200)
*Culex tritaeniorhynchus*	7500	75	12	1.60 (12/7500)
*Anopheles sinensis*	3300	33	4	1.21 (4/3300)
Total	12,000	120	18	1.50 (18/12000)

^†^RT-qPCR, quantitative reverse transcription polymerase chain reaction;

^‡^MIR, minimum infection rate. MIR uses the assumption that a positive pool contains only one infected mosquito the minimum infection rate, which is calculated: ([number of positive pools/total specimens tested] × 1000) (https://www.cdc.gov/westnile/resourcepages/mosqSurvSoft.html)

### Virus isolation and phylogenetic analyses

**Fig. 1:**
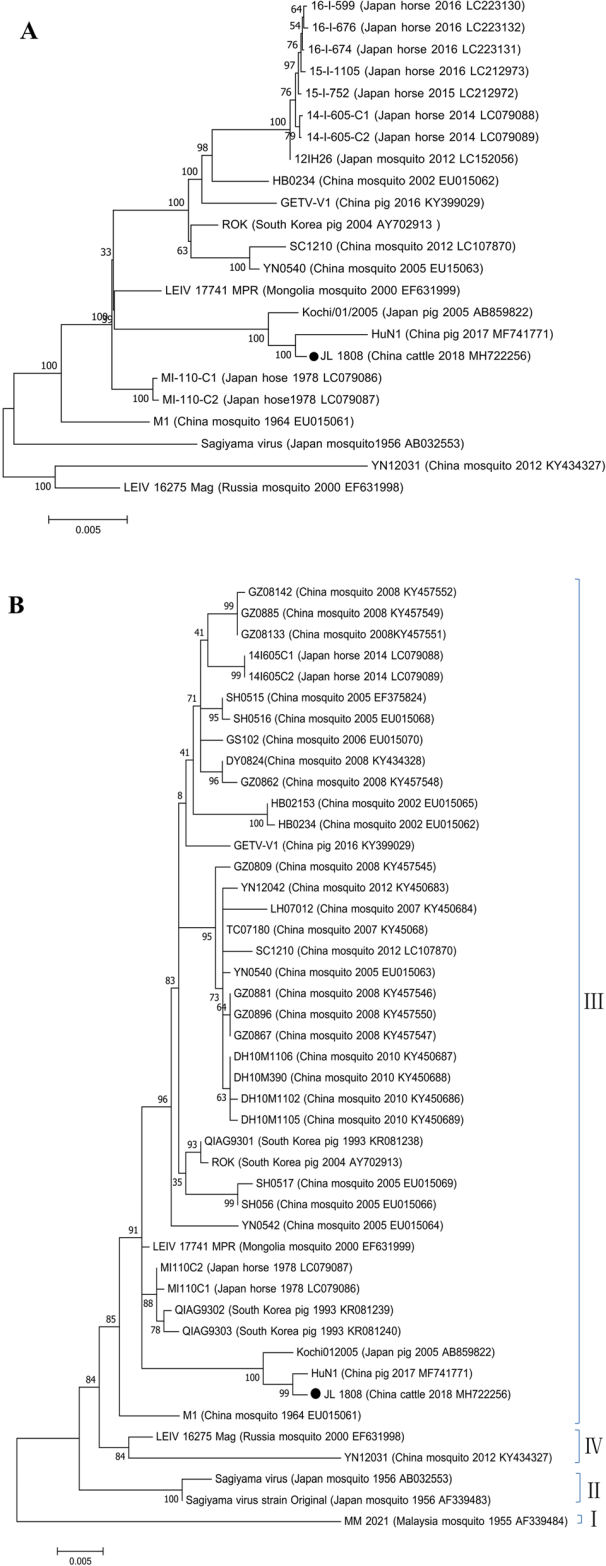
Phylogenetic analyses of the complete genome sequences (**a**) and E2 gene sequences (**b**) of Getah viruses isolated from cattle in Jilin, 2018. The evolutionary history was inferred using the maximum likelihood method with the Tamura-Nei model and gamma-distributed rate heterogeneity in MEGA version 7. The percentage of replicates in which the associated virus clustered together in the bootstrap test (1000 replicates) is shown next to the branch in each tree. The bootstrap support percentage is indicated by the value at each node. The strain isolated in this study is identified by black circles

A total of 48 cattle blood samples were used for virus isolation and inoculated in mouse neuroblastoma N2a cells (N2a) and Madin-Darby bovine kidney (MDBK) cell lines. Cytopathic effects (CPEs) were consistently observed in N2a and MDBK cells after four blind passages. Electron microscopic examination revealed spherical enveloped viral particles averaging 70 nm in diameter, which is a typical morphology of *Alphavirus*. We first isolated GETV from infected cattle and named the strain JL1808. The JL1808 strain (GenBank accession no. MH722256) genome contains 11,689 bp and encodes the non-structural polyprotein NS1–4 and the structural polyproteins capsid, 6 K and envelope 1–3, similar to other GETVs from pigs and horses. Sequence comparisons showed high identity with the swine epidemic strain (HuN1) at the nucleotide level (99.5%) and at the deduced amino acid level in non-structural polyproteins (99.7%) and structural polyproteins (99.9%), respectively (Table 3). Phylogenetic analysis of the complete genome and E2 gene revealed that the JL1808 strain was most similar to the recent epidemic HuN1 strain [9] (Fig. 1 a and b).

**Table 3:** Nucleotide and amino acid sequence and identity analyses of JL1808 and the other GETV strains

Virus isolates	JL1808 (%)				
	Complete genome	Non-structural polyprotein		Structural polyprotein	
	nt	nt	aa	nt	aa
14-I-605-C1	97.8	97.7	99.4	97.8	99.5
14-I-605-C2	97.8	97.7	99.4	97.7	99.5
15-I-752	97.8	97.6	99.3	97.7	99.5
15-I-1105	97.7	97.7	99.2	97.7	99.4
16-I-599	97.7	97.7	99.3	97.7	99.4
16-I-674	97.7	97.6	99.2	97.7	99.4
16-I-676	97.7	97.6	99.2	97.7	99.4
GETV-V1	97.9	97.9	99.4	97.7	99.4
HB0234	97.9	97.9	99.2	97.7	99.2
HuN1	99.5	99.4	99.7	99.7	99.9
Kochi/01/2005	99.6	99.6	99.7	99.6	99.7
LEIV 16275 Mag	97.5	97.6	99.4	97.3	99.1
LEIV 17741 MPR	98.6	98.5	99.5	98.6	99.6
M1	98.0	98.1	99.2	97.7	98.5
MI-110-C1	98.6	98.6	99.7	98.5	99.5
MI-110-C2	98.6	98.6	99.1	98.5	99.6
ROK	98.2	98.2	99.6	98.1	99.5
Sagiyama virus	97.3	97.5	99.3	96.8	98.3
SC1210	97.8	99.0	99.5	97.6	99.4
YN0540	98.0	97.9	99.5	97.9	99.5
YN12031	96.3	96.4	98.9	96.2	98.3

## Discussion

GETV has a broad geographical distribution and strong host adaptability. Neutralizing antibodies or nucleic acid sequences have been detected in pigs, horses, foxes, hamsters, chickens, ducks, dairy cattle, beef cattle, monkeys and humans [7, 11, 14, 16–18]. GETV has caused several disease outbreaks in pigs and horses over the past 20 years [9–11]. In a previous report, GETV was considered an emerging mosquito-borne virus, and the positive rate of animal neutralizing antibodies was related to the prevalence of GETV [16]. The positive rate of GETV antibodies in beef cattle averaged 72% in Yunnan Province, but viral RNA was not detected [16]. This study is the first to detect GETV RNA and neutralizing antibodies in clinically infected cattle in northeastern China. The antibody titres of the beef cattle specimens ranged between 1:640 and 1:1280. A similar result involving high neutralizing antibody titres (1:640 to more than 1:2560) has been shown in beef cattle [16] specimens. However, we first detected GETV RNA and isolated the virus from low-titre (1:5) serum specimens from beef cattle. Moreover, we observed that when antibody titres were between 1:40 and 1:1280, the cattle had few clinical symptoms, and viral RNA could not be detected in samples with antibody titres ranging from 1:10 to 1:1280. Beef cattle are the commonly bred in forested areas in Jilin Province, where there are large numbers of mosquitoes and host animals. To clarify the role of vectors in virs transmission, mosquitoes were tested for GETV; the results suggested that the virus was transmitted from mosquitoes to cattle in the study region. Mosquito transmission may pose a potential risk to humans and livestock; however, the epidemiology of GETV in terms of its association with its vectors and hosts remains to be explored. In this study, few GETV-positive beef cattle had clinical symptoms; more extensive studies should be conducted to clarify the mechanisms underlying the clinical symptoms, such as bacteria, parasites, poisoning or nutritional diseases, in the affected cattle.

In this study, GETV RNA and neutralizing antibodies were detected simultaneously for the first time in beef cattle, showing that the GETV host range has expanded to cattle. GETVs have evolved into four distinct evolutionary populations; however, only Group III includes all epidemic GETV strains that infect animals such as pigs and horses [12]. A phylogenetic analysis of the E2 gene indicated that the JL1808 strain was also clustered with Group III. Within the GETV Group III branch, JL1808 is evolutionarily related to the Chinese swine strain (HuN1), which caused approximately 200 piglets to die and more than 150 pregnant sows to produce stillbirths or foetal mummies in Hunan in 2017. Group III has completely replaced the original GETV populations (Groups I and II) in terms of geographical distribution, mosquito vectors and host animals [12]. Further long-term epidemiological surveys considering migratory birds, other animals and humans are needed. Moreover, an understanding of the natural circulation of GETV among domestic animals, especially pigs and cattle, is needed to prevent outbreaks of GETV in northeastern China.

## Conclusion

This is the first study to isolate GETV (JL1808) from cattle in northeastern China; the virus showed high identity to the China swine strain (GETV/HuN1) at the nucleotide and deduced amino acid levels. The phylogenetic analysis revealed that JL1808 was most similar to the HuN1 strain, which caused high rates of piglet mortality, stillbirths and foetal mummies in southern China in 2017. Moreover, GETV was detected in various species of mosquitoes in the studied areas. Our results indicate that GETV is transmitted by mosquitoes and may potentially pose a threat to animals in this area.

## Methods

### Collection of samples

Starting in 2018, 48 beef cattle blood samples were random collected from cattle in the forest grazing areas of Jiaohe (latitude: 43°83′-43°86′N; longitude: 127°27′-127°35′E), Jilin Province, northeastern China; 10 out of the 48 cattle presented fever (ranging from 39.5–42.0 °C), appetite loss, and depression. Approximately 12,000 mosquitoes were collected using ultraviolet lamps as attractants in this region. The mosquitoes were sorted by species and pooled, with approximately 100 mosquitoes/pool. The three dominant species were *Cx. tritaeniorhynchus* (75 pools), *An. sinensis* (33 pools), and *Armigeres subalbatus* (12 pools). Serum and mosquito samples were used to prepare 10% (v/v) or 10% (w/v) emulsions using Eagle’s minimum essential medium with 2% heat-inactivated foetal bovine serum. The samples were centrifuged at 12000×g for 30 min at 4 °C. The serum and mosquito supernatants were then passed through 0.22-μm syringe filters. After filtration, the supernatants were transferred to fresh microtubes and stored at − 80 °C until use in the experiments.

### Virus detection

To identify the possible causes of illness, the serum samples from cattle with clinical symptoms were pooled for viral metagenomic analyses as previously described [19]. To further validate the presence of GETV, viral RNA was extracted using a QIAamp Viral RNA Mini Kit (Qiagen, USA). The RNA was converted into cDNA using a Vazyme HiScript II 1st Strand cDNA Synthesis Kit (Vazyme Biotech Co., Ltd., China) in accordance with the manufacturer’s instructions. RT-qPCR was performed with RNA from the cattle serum and mosquito samples as described elsewhere [20]. Serum neutralization (SN) tests were carried out using the microtiter method as previously described [21].

### Virus isolation

Serum supernatants were diluted 20-fold in DMEM before inoculation onto N2a and MDBK cell monolayers for 1 h. After inoculation, the cell monolayers were washed twice with PBS and maintained in DMEM supplemented with 2% foetal calf serum (Gibco, USA), 100 U/ml penicillin and 100 μg/ml streptomycin at 37 °C in a 5% CO_2_; several blind passages were made until CPEs were observed.

### Electron microscopic analysis

The N2a and MDBK cells at 4 days post-infection were used for electron microscopic analysis. Cell supernatants were centrifuged at 12000×g for 5 min at 4 °C. Virus-containing supernatants were negatively stained and examined using transmission electron microscopy (TEM) [22].

### GETV complete genome determination

The complete genome of the novel GETV strain was obtained using a previously described method [12]. The PCR products were examined by agarose gel electrophoresis, purified using a QIAquick Gel Extraction Kit (Qiagen, USA) and sequenced.

### Genetic analysis of GETV

The complete genome and the E2 genes of the cattle GETV strain were aligned and phylogenetically compared with the sequences of other strains [9]. Multiple sequence alignments and sequence similarities were determined using DNA Star software. The maximum likelihood (ML) method was used to construct a phylogenetic tree in MEGA version 7.0. The reliability was evaluated by a bootstrapping analysis with 1000 replicates, and a bootstrap value more than 50% was considered significant.

## Additional file

Additional file 1: **Table S1.** Contigs of GETV in beef cattle by metagenomic analysis and their identities to the strain HuN1 (MF741771.1) (ZIP 18 kb) (DOCX 21 kb)

## Data Availability

The datasets used and/or analysed during the current study are available from the corresponding author upon reasonable request.

## Abbreviations

*An. Sinensis*: *Anopheles sinensis*
*Cx. pseudovishnui*: *Culex pseudovishnui*
*Cx. tritaeniorhynchus*: *Culex tritaeniorhynchus*
GETV: Getah virus
MIR: Minimum infection rate
ML: Maximum likelihood
RT-qPCR: Quantitative RT-PCR
