# Supplementary material for: First isolation and characterization of Getah virus from cattle in northeastern China
Source: BMC Vet Res. 2019 Sep 5;15:320. doi: 10.1186/s12917-019-2061-z (PMC6729113; doi:10.1186/s12917-019-2061-z)
Supplement: Supplementary file 1 — Table S1. Contigs of GETV in beef cattle by metagenomic analysis and their identities to the strain HuN1 (MF741771.1) (ZIP 18 kb) (DOCX 21 kb) [file 12917_2019_2061_MOESM1_ESM.docx]

Table S1 Contigs of GETV in beef cattle by metagenomic analysis and their identities to the strain HuN1 (MF741771.1)

| Contig | Location | Sequence (5’→3’) | Identity % |
| --- | --- | --- | --- |
| 1 | 327-624 | GAAAAGTGCGGAAGACCCAGAGAGGCTGGCGAATTACGCTCGAAAGCTGGCGAAAGCATCGGGGACTGTGCTAGACAAGAATGTGTCCGGAAAGATAACGGACCTGCAAGACGTCATGGCCACTCCAGACTTGGAATCCCCGACTTTTTGCCTGCACACTGACGAGACGTGCCGCACTAGGGCTGAGGTCGCCGTGTACCAGGACGTATACGCTGTGCACGCACCGACGTCTCTGTACCACCAGGCCATCAAAGGTGTCAGGACGGCGTATTGGATTGGATTTGACACCACTCCATTC | 100 |
| 2 | 34-155 | CTAGGATCCTTTGCTACTCCACATAGTGAGAGACAAACAACCCAAATGAAGGTAACCGTGGACGTTGAGGCTGATAGCCCATTCCTTAAGGCCCTTCAGAAGGCGTTTCCCGCCTTTGAGGT | 99 |
| 3 | 851-994 | ATTTACCTTCCGTGTTTCACCTGAAGGGTAAGAATAGTTTTACCTGCAGGTGCGACACGGTGGTGTCATGCGAAGGTTACGTGGTAAAGAAGATCACCATAAGCCCAGGCATATATGGAAAAACAGTCGATTACGCAGTTACCC | 99 |
| 4 | 1120-1332 | GACGTGACACCAGAGGATGCCCAGAAGCTCCTGGTTGGATTGAACCAACGCATAGTGGTGAATGGTAGAACGCAAAGAAACACAAACACAATGAAAAACTACCTACTGCCAGTGGTAGCGCAAGCATTCAGTAAATGGGCACGAGAGGCGCGCGCAGACATGGAGGATGAGAAACCCCTAGGCACCAGAGAACGCACGTTGACGTGTTGTTGC | 99 |
| 5 | 1288-1518 | GAGAAACCCCTAGGCACCAGAGAACGCACGTTGACGTGTTGTTGCCTGTGGGCGTTTAAAAGCCACAAAACCCACACCATGTACAAGCGGCCTGAAACGCAAACTATCGTCAAAGTGCCTTCCACTTTTGATTCCTTTGTGATACCGAGCCTGTGGTCATCCAGTCTTTCCATGGGTATCAGACAGAGAATCAAACTGCTACTCAGCGCAAGAATGACCCAAGGCCTACCA | 100 |
| 6 | 1730-1935 | ACGACCACCTCATAGGCTCCTACTTGATCCTTTCCCCCCAAACGGTGTTGAAAAGCGAGAAGCTGGCACCCATCCACCCTCTTGCTGAGCAAGTCACGGTCATGACCCACTCCGGAAGATCCGGTCGATACCCAGTCGACAAGTACGACGGACGGGTATTGATCCCAACAGGAGCGGCCATCCCAGTGAGCGAGTTCCAGGCACTC | 99 |
| 7 | 2025-2326 | CGACGAGGAAAGCTATGAAAAAGTGAGAGCTGAGAGGGCAGAGACAGAGTATGTGTTTGACGTGGACAAGAAGGCATGTATCAAGAAGGAGGAGGCATCAGGCCTTGTGTTAACAGGGGACCTAATCAATCCACCTTTCCACGAATTCGCATACGAAGGACTCAAGATCCGCCCAGCAGCCCCGTACCACACGACGATCATTGGTGTGTTTGGCGTTCCAGGTTCGGGCAAGTCGGCTATCATTAAGAACATGGTGACGACTCGCGATCTGGTGGCCAGTGGAAAGAAGGAGAACTGCCAAG | 99 |
| 8 | 2590-2853 | GTGCTCCATAAGAGCATCTCCAGAAGATGCACTCTACCTGTTACGGCGATCGTGTCCACCTTGCACTACCAAGGGAAGATGAGAACGACGAACCGATGCAACACCCCCATCCAGATTGACACCACCGGTTCCTCCAAACCAGCCTCAGGAGATATCGTGTTAACGTGCTTCCGCGGCTGGGTGAAGCAACTGCAAATAGACTATCGTGGACACGAGGTGATGACTGCAGCTGCTTCCCAGGGTTTGACAAGGAAAGGCGTGTAC | 99 |
| 9 | 3053-3074 | TGAGAGTGTTGAACGAGCGTCCAGCGGAGGTTGATCCATTCCAAAACAAGGCTAAAGTGTGCTGGGCAAAATGTCTGGTGCAAGTTCTTGAGACGGCCGGAATACGTATGACGGCAGATGAATGGAACACCATCTTGGCTTTCAGAGAGGACAGAGCGTACTCGCCAGAAGTCGCTCTCAACGAGATTTGCACCCGTTACTACGGCGTTGACCTAGACAGCGGCCTATTCTCAGCGCAGTCAGTTTCCCTCTTTTATGAGAACAACCACTGGGACAACAGGCCTGGAGGACGCATGTACGGGTTCAACCATGAAGTAGCCAG | 99 |
| 10 | 3373-3661 | AGGAAATACGCAGCCAGATTTCCTTTTCTACGTGGCAACATGAACTCGGGGCTGCAACTAAACGTCCCTGAGAGGAAGCTCCAACCTTTTAGCGCCGAATGCAACATAGTCCCATCCAATCGTCGGTTACCGCATGCTCTGGTCACTAGTTATCAGCAGTGCCGTGGAGAGAGGGTAGAGTGGTTGCTGAAAAAGATTCCAGGTCACCAAATGTTACTTGTAAGTGAGTACAACCTGGTGATACCTCACAAAAGAGTCTTCTGGATTGCACCTCCGCGGGTGTCAGGCG | 99 |
| 11 | 3874-4079 | GTCAGCGAGATGGTGGTGACAGCCCTGGCTAGGAAATTCTCGGCGTTCCGTGTCCTGAGACCGGCGTGCGTGACGAGCAACACAGAAGTGTTCCTGCTGTTTTCTAACTTTGATAACGGCAGAAGAGCGGTAACCTTGCACCAAGCTAACCAGAAACTTAGCTCAATGTATGCCTGCAACGGATTGCACACTGCTGGCTGTGCACC | 100 |
| 12 | 4263-4467 | GACCGTAATCCACGCAGTGGGACCAAATTTCTCCACCGTAACAGAAGCCGAAGGGGACAGAGAGCTAGCGGCCGCGTACCGAGCTGTGGCTAGCATAATTAGCACCAACAACATAAAGAGCGTCGCAGTACCGCTGCTGTCCACAGGCACCTTTTCTGGCGGTAAGGACAGAGTGATGCAGTCCTTGAACCACTTATTCACGGCATTG | 100 |
| 13 | 4831-5034 | TCGTCTACGCCGCCGAAAACGGTGCCGTGTCTATGTCGGTACGCGATGACCGCGGAGCGGGTTGCCAGACTTAGGATGAATAACACCAAAAACATCATCGTGTGCTCCTCCTTCCCATTGCCGAAGTACAGGATAGAAGGAGTGCAGAAGGTGAAGTGTGACCGAGTGCTAATTTTTGACCAGACCGTCCCGTCACTAGTAAGT | 99 |
| 14 | 5073-5352 | TAATGTGAGCCTGACTTCCACGACGTCGACGGGATCCGCATGGTCATTTCCATCGGAAACGACCTACGAAACTATGGAGGTCGTAGCCGAGGTACACACCGAACCTCCGATCCCTCCGCCTCGCCGACGTAGAGCAGCCGTCGCCCAACTTAGACAGGATCTGGAAGTCACCGAGGAGATCGAGCCTTACGTGACACAGCAAGCAGAGATCATGGTCATGGAGAGGGTCGTGACGACAGACATACGCGCTATCCCAGTCCCGGCACGGCGGGCCATTACA | 99 |
| 15 | 5387-5615 | CTACCGAACCTCCATCAGAACCGGAAGCTCCTATCCCGGCACCAAGAAAGAGAAGAACCACTAGCACCTCACCTCCGCATAACCCAGAGGATTTCGTTCCCAGGGTACCGGTTGAGTTACCGTGGGAGCCGGAAGACCTAGACATCCAATTCGGTGACTTGGAGCCACGCCGTCGGAACACCAGGGACCGAGATAGCAGCACAGGAATACAGTTCGGTGATATCGACTT | 99 |
| 16 | 5862-6067 | GAAGGCACTAATTGTAGAAAGACTACGCGAAGGAGCAAAGTTGTACCTCCATGAGCAAACCGACAAAGTACCCACATACACCAGCAAGTACCCTAGACCTGTGTACTCACCATCGGTGGATGACAGCCTGAGCGATCCGGAAGTGGCAGTGGCCGCCTGTAACTCTTTCTTAGAGGAGAATTATCCGACCGTGGCGAACTACCAGA | 100 |
| 17 | 6235-6519 | AACGTACTAGCCGCGGCCACCAAAAGAAATTGTAATGTCACCCAAATGAGAGAATTACCAACCATGGACTCTGCGGTGTTCAACGTAGAAAGCTTCAAAAAATACGCATGTACCGGCGAATATTGGCAAGAATTTAAAGACAATCCTATACGGATCACCACCGAAAACATAACGACGTACGTGGCTAAACTCAAGGGTCCTAAGGCTGCTGCCCTTTTTGCCAAGACGCATAACCTGGTGCCGCTTCAGGAGGTGCCAATGGACCGCTTCGTGATGGATATGAAG | 99 |
| 18 | 6590-6835 | CGGAACCATTGGCCACGGCATATCTATGCGGAATCCACAGAGAGTTAGTCAGGCGGCTAAAAGCCGTTCTGACCCCGAACATTCACACTCTGTTTGACATGTCGGCGGAGGACTTTGATGCCATCATAGCGGCACATTTCCAACCGGGAGATGCTGTACTGGAGACAGATATCGCATCCTTCGACAAGAGCCAGGACGACTCCTTAGCGCTAACGGCGTTAATGCTTCTGGAAGACCTCGGGGTCG | 99 |
| 19 | 6991-7220 | CGCGTCTTACGCGACAAATTATCGTCCTCGGCGTGCGCCGCCTTCATAGGCGATGACAACATAGTGCACGGCGTGAGGTCAGACCCGCTAATGGCAGAAAGGTGTGCGAGTTGGGTCAACATGGAAGTGAAGATCATCGATGCCACAATGTGTGAGAAACCACCATACTTTTGTGGTGGATTCATCCTGTACGACAGTGTCGCCGGTACAGCGTGTAGGGTTGCAGACCCACTA | 99 |
| 20 | 7342-7623 | TTAGACGTGGCACTGAGCTCAAGATACCAAGTCAGTGGCGTCGGAAACATCACTAGAGCGATGTCCACGCTGTCTAAGAATTTGAAGTCTTTTAGGAAAATAAGAGGTCCCATCATACATCTGTACGGCGGTCCTAAATAGATGCAGGATTACACTACATCTAAAGACCACGTATTACAGACATCATGAATTACATCCCAACTCAAACCTTTTACGGACGCCGTTGGCGACCACGCCCGGCGTACCGTCCATGGCGGGTGCCGATGCAGCCGGCCCCACCCA | 100 |
| 21 | 7731-7949 | AAGCCAAAGAAGAAGCCACAAAAAGCGAAGGCTAAGAAAAACGAACAGCAAAAGAAAAACGAGAACAAGAAACCACCACCTAAGCAGAAGAATCCGGCTAAGAAGAAGAAACCAGGAAAAAGGGAACGCATGTGCATGAAGATAGAGAATGATTGCATCTTCGAGGTCAAGCTTGACGGTAAGGTCACGGGCTACGCCTGCCTAGTCGGGGATAAAGTG | 100 |
| 22 | 8132-8398 | GTACAGCGGTGGCAGGTTCACAATCCCGACAGGCGCAGGTAAACCGGGAGACAGCGGCCGGCCGATCTTCGACAACAAAGGACGTGTGGTGGCCATTGTCCTGGGAGGGGCCAACGAAGGAGCCAGGACTGCCCTATCTGTCGTGACCTGGACCAAAGACATGGTCACACGGTACACCCCAGAAGGAACAGAAGAATGGTCCGCCGCCTTGATGATGTGCGTCTTAGCCAACGTTACATTCCCATGCTCAGAGCCCGCATGTGCACC | 99 |
| 23 | 8624-8907 | AGAAAAAATTAGGGATGAGGCTTCCGATGGCATGATAAAGATCCAGATCGCAGCGCAAATCGGCATCAACAAAGGAGGAACACACGAACACAACAAAATCAGGTACATCGCTGGGCATGACATGAAAGAGGCGAACCGGGATTCTTTACAAGTGCATACTTCCGGTGTGTGCGCTATCCGAGGCACGATGGGCCACTTCATCGTGGCCTACTGCCCTCCAGGGGACGAACTAAAGGTCCAGTTCCAAGATGCAGAATCGCACACACAGGCCTGCAAAGTGCAGT | 99 |
| 24 | 9028-9391 | TGCATACCCCACCGGATATCCCAGATATAACGTTGCTGTCGCAGCAGTCAGGTAATGTAAAGATCACAGCAGGAGGAAAGACCATCAGATACAACTGCACGTGCGGTAGTGGCCATGTGGGCACCACCAGTAGCGACAAGACTATCAATTCGTGCAAAATAGCGCAGTGCCACGCTGCGGTGACTAACCACGACAAGTGGCAGTACACCTCCTCGTTTGTCCCTAGAGCCGACCAGTTGTCTCGCAAAGGTAAAGTGCACGTACCCTTCCCTCTGACCAACTCCACATGCAGGGTGCCCGTTGCACGTGCACCAGGTGTCACATACGGAAAGAGAGAACTGACAGTGAAACTGCACCCAGATCA | 100 |
| 25 | 9582-9782 | ATCATACTCTATTACTATGGGCTATACCCAGCAGCCACCATCGCCGCCGTCTCAGCCGCGGGTCTCGCAGCCATATTATCGCTGCTGGCGTCATGTTACATGTTCGCCACTGCACGCCGCAAGTGCTTGACCCCATACGCCTTGACCCCCGGGGCCGTCATTCCGGTAACACTAGGAGTACTATGCTGCGCGCCACGAGCG | 99 |
| 26 | 9973-10188 | ACGAACACACCGCAACGATCCCGAACGTGGTGGGGTTCCCGTATAAGGCTCACATTGAGAGGAACGGCTTCTCCCCGATGACCCTACAGCTTGAGGTACTTGGAACCAGCTTGGAACCCACGCTAAACTTAGAGTACATAACTTGCGAATACAAGACAGTCGTGCCATCACCTTATATCAAGTGCTGTGGGACATCAGAATGCAGATCCATGGAGC | 99 |
| 27 | 10340-10597 | GGCGCACACTGCGGCAATGAAAGCCACCATCCGAATAAGTTACGGGAACCTCAATCAGACAACAACGGCGTTCGTCAACGGGGAGCATACAGTGACCGTCGGAGGCAGCAGGTTTACTTTTGGTCCAATCTCCACTGCCTGGACGCCTTTCGACAACAAGATCGTCGTCTACAAGAACGACGTCTACAATCAGGACTTCCCACCCTACGGGTCAGGACAACCAGGGAGGTTCGGAGACATCCAGAGCAGGACGGTAGA | 99 |
| 28 | 10892-11116 | GCACTCATCGGACTTCGGCGGGATCGCAACTCTGACTTTCAAAACCGACAAACCCGGAAAATGTGCTGTCCATTCTCATTCGAATGTAGCCACCATACAGGAGGCAGCTGTGGACATCAAAACAGATGGCAAGATAACCCTGCATTTCTCTACAGCATCTGCATCCCCGGCATTCAAGGTATCTGTGTGCAGTGCCAAAACGACATGCATGGCAGCGTGTGAGCC | 99 |
| 29 | 11275-11410 | CTATGCGCCGCTAACCGGGAGGCTTGACATAATGTATATATATAAGCATCATAGTTTTAGTAAAGCATATAAATAATCAAGTAGATCAAAGGGCTACCTAACCCCTGAATAGTAACAAAACGCAAAATACAAAAACATTAGTTCAAAGGGCCAGTAACCCCTGAATAGTAACAAAACATAAAAACCAAAAACAGTAGTTCAAAGGGCTATACAACCCCTGAATAGTAA | 99 |
